# On the Origin of Reverse Transcriptase-Using CRISPR-Cas Systems and Their Hyperdiverse, Enigmatic Spacer Repertoires

**DOI:** 10.1128/mBio.00897-17

**Published:** 2017-07-11

**Authors:** Sukrit Silas, Kira S. Makarova, Sergey Shmakov, David Páez-Espino, Georg Mohr, Yi Liu, Michelle Davison, Simon Roux, Siddharth R. Krishnamurthy, Becky Xu Hua Fu, Loren L. Hansen, David Wang, Matthew B. Sullivan, Andrew Millard, Martha R. Clokie, Devaki Bhaya, Alan M. Lambowitz, Nikos C. Kyrpides, Eugene V. Koonin, Andrew Z. Fire

**Affiliations:** a Departments of Pathology and Genetics, Stanford University, Stanford, California, USA; b Department of Chemical and Systems Biology, Stanford University, Stanford, California, USA; c National Center for Biotechnology Information, National Library of Medicine, National Institutes of Health, Bethesda, Maryland, USA; d Skolkovo Institute of Science and Technology, Skolkovo, Russia; e Department of Energy, Joint Genome Institute, Walnut Creek, California, USA; f Department of Molecular Biosciences, Institute for Cellular and Molecular Biology, University of Texas at Austin, Austin, Texas, USA; g Department of Biomedical Informatics, Stanford University, Stanford, California, USA; h Department of Plant Biology, Carnegie Institution for Science, Stanford, California, USA; i Department of Microbiology, The Ohio State University, Columbus, Ohio, USA; j Departments of Molecular Microbiology and Pathology and Immunology, Washington University School of Medicine, St. Louis, Missouri, USA; k Department of Civil, Environmental and Geodetic Engineering, The Ohio State University, Columbus, Ohio, USA; l Department Infection, Immunity and Inflammation, University of Leicester, Leicester, United Kingdom; m Microbiology and Infection Unit, Warwick Medical School, University of Warwick, Warwick, United Kingdom; Columbia University

**Keywords:** CRISPR, RNA spacer acquisition, cyanobacteria, deep sequencing, horizontal gene transfer, host-parasite relationship, phylogeny, reverse transcriptase

## Abstract

Cas1 integrase is the key enzyme of the clustered regularly interspaced short palindromic repeat (CRISPR)-Cas adaptation module that mediates acquisition of spacers derived from foreign DNA by CRISPR arrays. In diverse bacteria, the *cas1* gene is fused (or adjacent) to a gene encoding a reverse transcriptase (RT) related to group II intron RTs. An RT-Cas1 fusion protein has been recently shown to enable acquisition of CRISPR spacers from RNA. Phylogenetic analysis of the CRISPR-associated RTs demonstrates monophyly of the RT-Cas1 fusion, and coevolution of the RT and Cas1 domains. Nearly all such RTs are present within type III CRISPR-Cas loci, but their phylogeny does not parallel the CRISPR-Cas type classification, indicating that RT-Cas1 is an autonomous functional module that is disseminated by horizontal gene transfer and can function with diverse type III systems. To compare the sequence pools sampled by RT-Cas1-associated and RT-lacking CRISPR-Cas systems, we obtained samples of a commercially grown cyanobacterium—*Arthrospira platensis*. Sequencing of the CRISPR arrays uncovered a highly diverse population of spacers. Spacer diversity was particularly striking for the RT-Cas1-containing type III-B system, where no saturation was evident even with millions of sequences analyzed. In contrast, analysis of the RT-lacking type III-D system yielded a highly diverse pool but reached a point where fewer novel spacers were recovered as sequencing depth was increased. Matches could be identified for a small fraction of the non-RT-Cas1-associated spacers, and for only a single RT-Cas1-associated spacer. Thus, the principal source(s) of the spacers, particularly the hypervariable spacer repertoire of the RT-associated arrays, remains unknown.

## IMPORTANCE

While the majority of CRISPR-Cas immune systems adapt to foreign genetic elements by capturing segments of invasive DNA, some systems carry reverse transcriptases (RTs) that enable adaptation to RNA molecules. From analysis of available bacterial sequence data, we find evidence that RT-based RNA adaptation machinery has been able to join with CRISPR-Cas immune systems in many, diverse bacterial species. To investigate whether the abilities to adapt to DNA and RNA molecules are utilized for defense against distinct classes of invaders in nature, we sequenced CRISPR arrays from samples of commercial-scale open-air cultures of *Arthrospira platensis*, a cyanobacterium that contains both RT-lacking and RT-containing CRISPR-Cas systems. We uncovered a diverse pool of naturally occurring immune memories, with the RT-lacking locus acquiring a number of segments matching known viral or bacterial genes, while the RT-containing locus has acquired spacers from a distinct sequence pool for which the source remains enigmatic.

## INTRODUCTION

Clustered regularly interspaced short palindromic repeat (CRISPR)-Cas systems provide adaptive immunity in most of the archaea and many bacteria (1). Short genomic segments of invading genetic elements (protospacers) are inserted as spacers between direct repeats in the CRISPR array(s) of the host genome during CRISPR adaptation (2–4). The primary transcript of the CRISPR array is processed into small-RNA guides that consist of a spacer with portions of the flanking CRISPR repeats, which are used by CRISPR-associated (Cas) nucleases to identify and degrade cognate nucleic acids (5, 6). The information content of CRISPR spacers is integrated directly into the prokaryotic genome and is inherited upon cell division, creating a durable genomic record of a host-pathogen interaction.

CRISPR-Cas systems have been classified into six types and more than 20 subtypes on the basis of gene content and locus architecture (7–9). CRISPR repeat sequences of the various types and subtypes show conservation in related bacteria, while the spacer content of the CRISPR arrays is extremely variable (10). Genomes of different strains of the same bacterial species often contain similar configurations of CRISPR-Cas systems with closely similar repeat sequences but substantially different and, in many cases, non-overlapping spacer repertoires (11).

Several type III CRISPR-Cas systems contain genes encoding fusions of reverse transcriptases (RTs) with the major CRISPR adaptation enzyme, the Cas1 integrase (12–15). Here, we explore the possible origins of RT-associated CRISPR loci, the routes of their evolution, and their spacer content. In particular, we address several open questions. When and how did putative functional associations between RTs and *cas1* genes arise? What constraints exist for the association of RT-Cas1 fusions with CRISPR-Cas systems? Do RT-Cas1-containing CRISPR loci acquire spacers from a distinct source, i.e., from genomic RNA, plasmid RNA, DNA phage transcripts, or RNA phage sequences, or from some other, uncharacterized RNA pool?

We have previously shown that RT-Cas1 fusions enable CRISPR spacer acquisition directly from RNA (16). The ability of the RT-Cas1 adaptation modules to acquire spacers from RNA, coupled with the ability of type III CRISPR-Cas effector modules to target RNA (17–24), implies the potential to provide adaptive immunity against parasites with both RNA and DNA genomes. We wondered whether such a capability might lend itself to an expanded immune function for RT-associated CRISPR-Cas systems in the environment.

The population of spacers contained within CRISPR arrays in a natural community of RT-Cas1-carrying organisms is a potential “memory bank” of the pools of nucleic acids, particularly RNAs, that are perceived as “threats” by these microbes (25). To gain insight into this form of microbial immune memory, we interrogated the spacer repertoire of a natural population of RT-Cas1-bearing cyanobacteria *Arthrospira platensis* grown in vast enriched cultures for commercial sale as a food supplement known as *Spirulina*. The *A. platensis* genome contains both an RT-linked type III-B CRISPR-Cas locus and an RT-lacking type III-D locus. We identified diverse spacer repertoires from each CRISPR-Cas system. Protospacers were identified in the *Spirulina* metagenome for a few type III-D spacers, but virtually none of the RT-linked type III-B spacers could be traced to any source. These findings suggest that the vast majority of RT-linked CRISPR spacers come from a distinct sequence pool, the nature of which remains enigmatic.

## RESULTS

### Phylogeny of reverse transcriptases associated with CRISPR-Cas systems.

**FIG 1:**
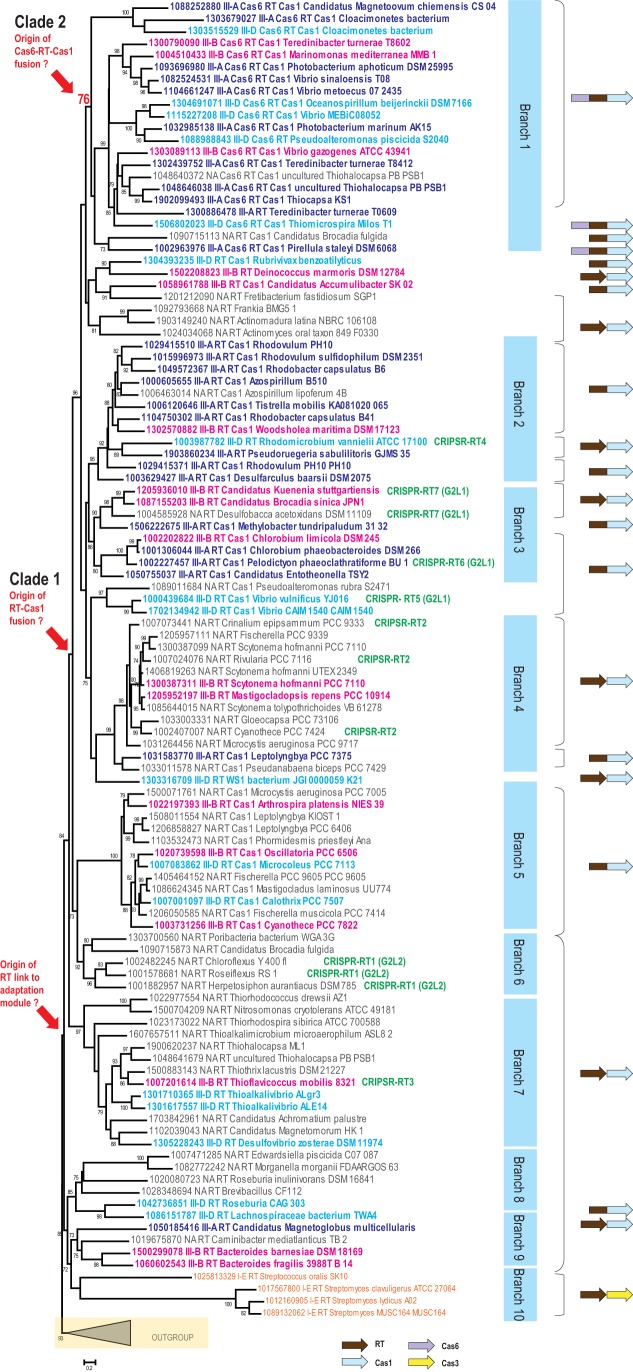
Phylogeny of a representative set of reverse transcriptases encoded within CRISPR-*cas* loci. A maximum likelihood phylogenetic tree was reconstructed for 134 RT sequences using the FastTree program. SH (Shimodaira-Hasegawa)-like node support values calculated by the same program are shown if they are greater than 70%; node support values for key nodes are highlighted. Major well-supported distinct branches are shown by blue rectangles. Each sequence in the tree is shown with a local numeric identifier (ID) and species name; these are also provided in Table S1 (https://figshare.com/s/3a8dab8ed7138922f693) for comparison. RT protein domain architecture is coded in each sequence description as follows: Cas6_RT_Cas1 and RT_Cas1 for the respective fusions, RT for the systems with known subtypes, and NA_RT for all other cases. A typical domain or gene organization for each branch and for selected sequences is shown to the right of the tree. Independent genes are shown with distinct arrows, while fused genes are displayed as single arrows with multiple colors. The text is color-coded to denote CRISPR-Cas system subtypes as follows: III-A, dark blue; III-B, magenta; III-D, sky blue; I-E, orange. The outgroup is collapsed and is indicated by a triangle. The details for the outgroup branch are provided in Fig. S1. For the sequences that were classified previously (15), the respective groups are indicated in green.

Previous phylogenetic analysis of the RT superfamily has suggested that the CRISPR-associated RTs could be derived from a single acquisition event, and that they are most similar to the RTs encoded by group II introns (15). To study the origin and extent of the RT association with CRISPR loci in greater detail, we retrieved 266 islands from a large dataset of both complete and draft bacterial and archaeal genomes that contained at least one *cas* gene and at least one RT gene (see Table S1 at https://figshare.com/s/3a8dab8ed7138922f693). From this collection of genomic loci, we selected a nonredundant set of proteins that could be expected to contain a full-sized RT domain (~300 amino acids [aa]). As reported previously, the CRISPR-associated RTs do not show specific phylogenetic affinity to any subgroup of the group II intron-encoded RTs (15). To build a phylogenetic tree, we included several group II intron-encoded RTs as an outgroup (Fig. 1; also see Fig. S1 in the supplemental material for details). The resulting tree topology is largely consistent with the phylogenetic analysis in the previous work (15), with the seven distinct groups described in the previous analysis reproduced in our tree (Fig. 1). Because our dataset included almost an order of magnitude more RTs than were used in the previous set, we were able to identify additional, well-supported branches that had not been described previously. These new groupings include branch 1, which consists of proteins containing fused Cas6, RT, and Cas1 domains; the Cyanobacteria-specific branch 5, which consists of RT-Cas1 fusion proteins; minor branches 8, 9, and 10 (Fig. 1); and the Methanomicrobia-specific branch 11 in the outgroup (Fig. S1). The RT from *Marinomonas mediterranea*, for which activity in spacer adaptation has been studied (16), belongs to branch 1, and the RT from *A. platensis* belongs to branch 5 (Fig. 1).

10.1128/mBio.00897-17.2 FIG S1  Phylogeny of selected representatives of reverse transcriptases encoded in genomic regions shared with Cas genes: the outgroup branch (see Fig. 1). Designations are the same as those used in Fig. 1. In addition, group II intron-associated RTs are shown in brown and other CRISPR-Cas systems in purple. Comments pertaining to type III systems and RT-containing loci are provided in parentheses. Similarly to the RT-associated adaptation module, the RT-*cas6* gene pair is promiscuous and was identified within three distinct type III subtypes. The *cas1* genes associated with these systems are confidently placed within the subtree that includes the other RT-associated *cas1* genes. Thus, in this case, the RT from a group II intron most likely displaced the ancestral RT in a CRISPR-*cas* locus. This substitution was then followed by gene rearrangement and dissociation from the adaptation module. Download FIG S1, EPS file, 1.6 MB. Copyright © 2017 Silas et al. 2017 Silas et al. This content is distributed under the terms of the Creative Commons Attribution 4.0 International license.

To investigate the origin of the fusions between RT and *cas* genes, we examined the domain organizations of all Cas proteins fused to RT domains (or encoded by adjacent genes) in the context of the RT tree (Fig. 1). The tree suggests the original establishment of a functional connection between RT and *cas1* within the adaptation module, first as adjacent genes. The tree topology is further consistent with the parsimonious evolutionary scenario of single points of origin, first of the RT-Cas1 fusion and then of the Cas6-RT-Cas1 fusion (clades 1 and clade 2, respectively) (Fig. 1). However, there are many variations in protein architecture in different branches of the tree. Some of this variation in clades 1 and 2 can be explained by secondary fissions (i.e., split of the fusion protein into separate Cas1 and RT proteins), although the possibility of there being several additional, independent cases of RT-Cas1 fusion within or outside these clades (e.g., *Roseburia* in branch 8) cannot be ruled out. The Cas1 phylogeny largely follows the RT phylogeny (Fig. S2), which suggests that the two proteins (or domains in fusion proteins) generally coevolve. Recombination between RT and Cas1 domains could have occurred on several occasions and might account for the inconsistencies between the topologies of the RT and Cas1 trees (for example, see *Pirellula staleyi* DSM 6068 in Fig. 1 and Fig. S2).

10.1128/mBio.00897-17.3 FIG S2  Phylogeny of RT-linked Cas1 proteins. Designations are the same as those used in Fig. 1. Download FIG S2, EPS file, 2.8 MB. Copyright © 2017 Silas et al. 2017 Silas et al. This content is distributed under the terms of the Creative Commons Attribution 4.0 International license.

**FIG 2:**
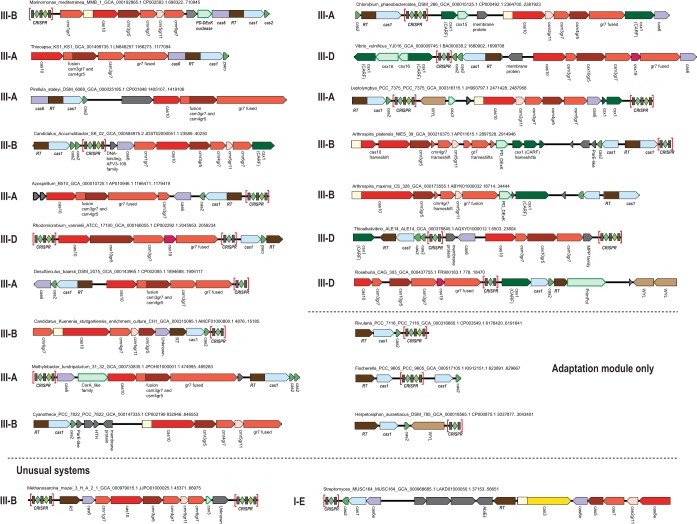
Architectures of selected RT-associated CRISPR-Cas loci. For each locus, the species name, genome accession number, and respective nucleotide coordinates are indicated. Genes are shown roughly to scale; CRISPR arrays are indicated in brackets and are not shown to scale. Homologous genes are color-coded, with the exception of numerous ancillary genes, which are all shown in light green with a green outline, and unknown proteins are shown in gray. The gene names largely follow the nomenclature in reference 7, but the RAMP proteins of groups 5 and 7 are denoted gr5 and gr7, respectively. The CRISPR-Cas system subtype is indicated for the loci encoding the respective effector genes.

We also mapped the known CRISPR-Cas system subtypes onto the RT tree to determine whether the RTs coevolved with the respective CRISPR effector complexes. The RT-Cas1 fusions were associated with diverse subtypes and variants of type III CRISPR-Cas systems (Fig. 1). On the whole, we observed virtually no link between the RT phylogeny and the phylogeny of the effector modules. Furthermore, even within the RT-encoding loci classified as being of the same CRISPR-Cas subtype, gene content was often notably different (Fig. 2). About half of the RT-*cas* loci lack genes for effector complex subunits but often include a CRISPR array (Fig. 2). Taken together, these observations indicate that the RT-containing adaptation module represents an autonomous functional unit that spreads by horizontal transfer and can promiscuously combine or function in *trans* with any type III system (with the possible exception of type III-C). It should be noted, however, that given the absence of the hallmarks of group II introns, including both the typical elements of RNA secondary structure and additional domains of the intron-encoded proteins (26), these RT-encoding modules are not predicted to spread as retroelements. With a single exception described below, RTs were not found associated with other CRISPR-Cas types.

In the course of this analysis, we detected two unusual RT-associated CRISPR-Cas architectures. One of these belongs to branch 11 in the outgroup (i.e., among group II intron-encoded RTs) and is present mostly in *Methanosarcina* species. In this case, the RT gene is located next to a *cas6* gene but there is no *cas1* gene in the locus (Fig. 2). This RT is confidently placed within the outgroup containing mostly RTs of group II introns (Fig. 1). Thus, this is most likely a case of independent recruitment of an RT into a CRISPR-Cas system. The second unusual architecture occurs in association with a small number of type I-E systems. The RT genes in this case are located next to the effector module genes (Fig. 2). This arrangement was detected in several *Streptomyces* species and in *Streptococcus oralis* (Fig. 1). The RT in this system seems to have been acquired from the main, type III-associated group described above (Fig. 1). The fixation of this gene arrangement in bacterial evolution implies that this RT functions as part of the type I-E CRISPR-Cas system, which is unexpected given the lack of RNA targeting by the type I systems that have been studied so far, and could be an interesting direction for experimental study.

### A diverse repertoire of CRISPR spacers in commercially grown *Arthrospira platensis.*

To compare the nucleic acid sequence repertoires sampled by RT-Cas1-associated and RT-lacking CRISPR-Cas systems in a physiological setting, we obtained a series of independent samples from the cyanobacterial species *Arthrospira platensis*, grown commercially in open-air “raceway” ponds and marketed as *Spirulina*. We chose this species because it represented an easily accessible, natural population of RT-Cas1-encoding bacteria, grown in a large culture exposed to the open environment.

**FIG 3:**
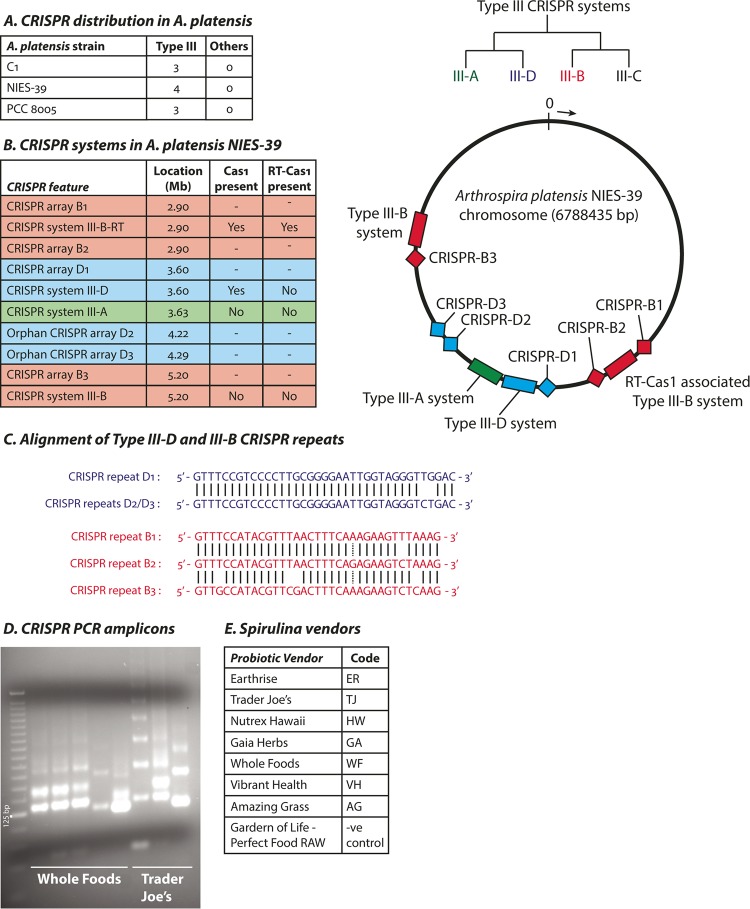
CRISPR-Cas systems in *Arthrospira platensis*. (A) Distribution of CRISPR-Cas systems by phylogenetic type in three sequenced reference strains of *A. platensis* ("Others": types I, II, IV, V, and VI). The tree at the right side of the panel shows the evolutionary relationship between type III subtypes. (B) List of CRISPR-Cas systems and arrays in type strain *A. platensis* NIES-39 (left panel). The approximate location in megabases (Mb) on the circular chromosome in the direction of the arrow is indicated (right panel). The *cas10 cmr2* and *cmr6* family genes in the III-B–RT system (but not the III-B system) show signs of mutational atrophy. (C) Alignments of CRISPR direct repeats from the various CRISPR arrays. (D) Gel image showing PCR products amplified from CRISPR arrays. The first lane shows a 25-bp DNA ladder. (E) List of *Spirulina* brands used in this study.

The various *A. platensis* strains with sequenced genomes (8005, C1, NIES-39) contain type III-A, type III-D, and type III-B CRISPR-Cas systems, and two classes of CRISPR arrays with conserved CRISPR repeat sequences that are associated with either the III-D or III-B systems. None of the strains harbor any type I or type II CRISPR-Cas systems. One type III-B locus (denoted III-B–RT) in each strain carries an RT-Cas1 fusion in addition to a separate Cas1 gene without an RT domain. Additionally, InsQ and COG2452 transposases, as well as apparent pseudogenes of IS*1*, IS*607*, and IS*630* family transposases and group II intron-type RTs, can be found in the immediate neighborhoods of the type III-B loci. The arrangement of various CRISPR-Cas systems in the type strain *A. platensis* NIES-39 is shown (Fig. 3A to C).

We designed primers specific to the distinct III-D (RT-lacking) and III-B (RT-Cas1-associated) CRISPR repeat sequences in the *A. platensis* NIES-39 genome to amplify spacers selectively from each CRISPR array (Fig. S3). Spacers are typically integrated into CRISPR arrays in a directional manner: new spacers are inserted close to the “leader” sequence (which also contains the CRISPR promoter) and are pushed down the array when more spacers are acquired (4). Older spacers would therefore be seen more frequently in a bacterial population as they are propagated through cell division. Our sequencing method retains strand information but should lead to the amplification of all spacers with similar efficiencies irrespective of the ordering of the spacers in a CRISPR array.

10.1128/mBio.00897-17.4 FIG S3  Spacer amplification PCR strategy. First, primers that bind to the CRISPR repeat sequences are used to amplify spacers from the array. A forward primer in any repeat may amplify a product in concert with a reverse primer with any subsequent repeat; however, the shortest (~125-bp) amplicons consisting of one CRISPR spacer are most efficiently amplified, while larger amplicons containing two, three, or more spacers produce the characteristic “ladder” pattern of PCR products shown in Fig. 3D. DNA adapters to make the amplicons compatible with Illumina high-throughput sequencing technology are attached in a subsequent round of PCR, and a final product corresponding to the ~125-bp amplicon from step 1 is size selected and sequenced. Download FIG S3, EPS file, 1.6 MB. Copyright © 2017 Silas et al. 2017 Silas et al. This content is distributed under the terms of the Creative Commons Attribution 4.0 International license.

We purchased *Spirulina* from various *Spirulina* vendors at local grocery stores and extracted genomic DNA from the cyanobacteria. This DNA served as the template for amplification of CRISPR spacers, and the amplicons formed the characteristic “ladder” expected of primers that bind only in the repeat sequences of CRISPR arrays (Fig. 3D and E). We also used as an amplification control a similarly marketed raw probiotic blend that does not contain *Arthrospira platensis* and did not observe any amplicons (data not shown).

**FIG 4:**
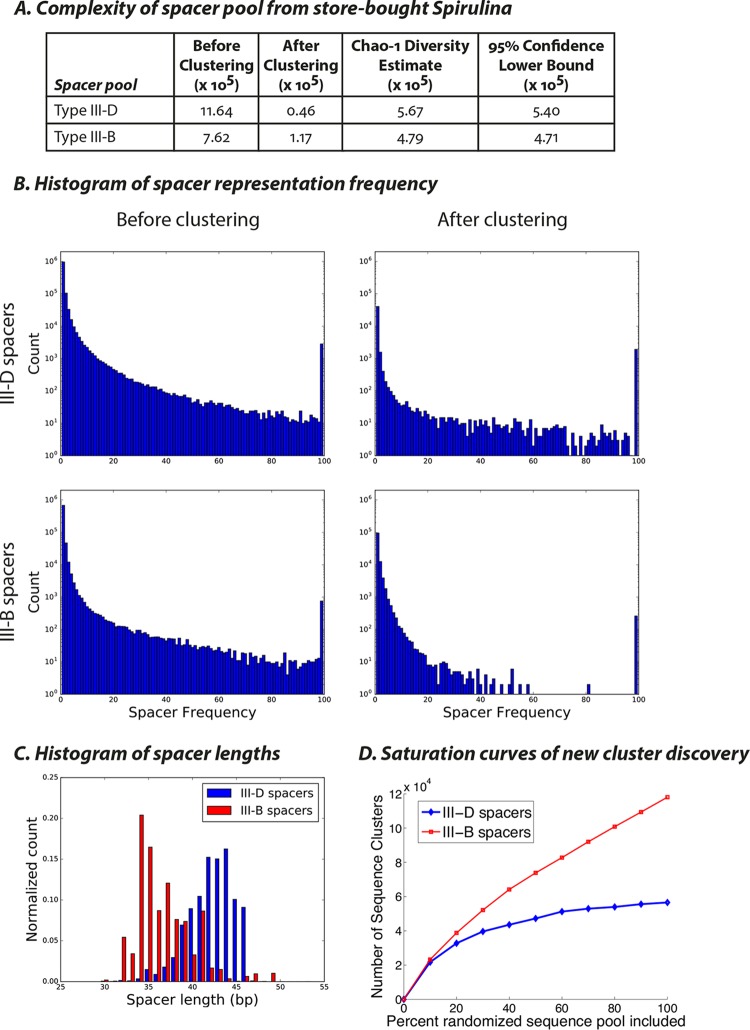
Sequencing of spacers from *Spirulina* purchased from grocery stores. (A) Numbers of unique spacers recovered from type III-D and type III-B CRISPR arrays before and after clustering. The last two columns show Chao-1 estimates of species richness/diversity (57). The Chao-1 estimate corresponds to an approximate lower bound for the total number of unique spacers in the sample. The 95% confidence lower bound for the Chao-1 estimate has also been calculated. (B) Histogram of spacer frequency before and after clustering. The last bin contains all sequences observed 100 times or more. (C) Histogram of spacer lengths for III-D and III-B spacers. (D) Saturation curves calculated for III-D and III-B spacer pools using the clustering algorithm described for panel A showing the number of sequence clusters obtained as progressively larger subsets of the spacer datasets were considered.

By high-throughput sequencing of the smallest amplicon from each reaction (corresponding to one repeat-spacer-repeat unit), we recovered a diverse repertoire of spacer sequences. To group highly similar spacers, we first trimmed CRISPR repeats from the reads and allowed up to 2 mismatches to account for sequencing error. This procedure yielded a total of about 2 × 10^6^ unique spacers. Variation in spacer sequences caused by mutations arising during cyanobacterial cell division could result in multiple “unique” spacers being identified that were likely derived from the same acquisition event. To obtain a more conservative estimate of the complexity of the spacer pools, we utilized a less stringent threshold for spacer equivalence. Spacers were ranked by the frequency with which they were encountered in the dataset and were then compared pairwise with every less prevalent sequence. If the two sequences were different at 4 or fewer sites (allowing for arbitrarily long deletions on either end to account for potential trimming artifacts), the less prevalent sequence was discarded. For every spacer observed 100 times or more, we also discarded any less frequent sequences that shared any 12-mer with their presumed “parents.” This procedure yielded a dataset of ~2 × 10^5^ spacer clusters (Fig. 4A). The clustered spacers were typically observed only once in the dataset (Fig. 4B).

The spacers from the III-B system were shorter than those from the III-D system, as expected from their sizes in the sequenced CRISPR arrays in the reference genome (Fig. 4C). Coincidence analysis (27, 28) revealed that spacers amplified from the III-D and III-B loci indeed came from distinct sequence pools (Fig. S4). We found the spacers from CRISPRs B1/B2, CRISPR B3, and CRISPRs D1/D2/D3 to be mutually disjoint. Further validating the specificity of our PCR method, spacers in the CRISPR arrays in the reference genomes of various *A. platensis* strains were efficiently recovered, and there were no instances of crossovers between the III-D and III-B spacer pools at the genomic CRISPR loci. To assess whether we have approached a point of diminishing returns in our sequencing efforts, we randomly sampled spacers from our dataset in 10% increments and quantified whether the total number of unique spacers (counted according to the clustering method described above) kept increasing. We found that the spacer pools appeared to be approaching diminishing returns for the type III-D loci but not for the type III-B loci (Fig. 4D).

10.1128/mBio.00897-17.5 FIG S4  Coincidence analysis of spacer sequencing datasets. To measure the pairwise similarity of the sequenced spacer replicates, we computed the pairwise coincidence probability (or co-clonality score) from the replicates. The coincidence probability (or co-clonality score) between two replicates is the probability that a random spacer from one replicate would happen to be the same as a random spacer from a second replicate. The logarithms of these probabilities are shown in a heat map. The heat map shows that the replicates organize themselves by CRISPR loci into clear clusters. Download FIG S4, EPS file, 2.3 MB. Copyright © 2017 Silas et al. 2017 Silas et al. This content is distributed under the terms of the Creative Commons Attribution 4.0 International license.

### Sampling the extracellular environments of *A. platensis.*

We reasoned that the appropriate environment in which to find the sequence pool from which *A. platensis* captures spacers would be open-air “raceway ponds”. We collected samples of the cyanobacteria and their extracellular environment from a commercial *Spirulina* farm (Earthrise Nutritionals LLC, Calipatria, CA). Several samples of enriched culture were collected directly from the *Spirulina* raceway ponds and separated into cellular and extracellular fractions by centrifugation (see Materials and Methods). A summary of the sequencing data thus obtained is shown in Fig. S5A. Mapping of the DNA sequences from the cellular fraction to the *A. platensis* reference genomes (strains NIES-39, C1, and 8005) revealed a highly polymorphic population (Fig. S5B). Because we were most interested in potentially rare and/or structured RNA species, the isolated RNA was sequenced using several different methods to mitigate inherent biases among the RNA sequencing protocols (see Materials and Methods). Virus-like contigs were detected in both extracellular RNA and DNA fractions (Fig. S5C and D; also see Text S1 in the supplemental material). In addition to the data from *Spirulina* raceway ponds, small amounts of metagenomic data were collected from the nearby saline rift lake, the Salton Sea. We also obtained a metagenomic DNA dataset from a separate study of Lake Bogoria in Kenya (unpublished); this lake is a natural habitat of cyanobacteria, including *A. platensis*, which serve as the food source for one of the world’s largest populations of flamingos.

10.1128/mBio.00897-17.6 FIG S5  Metagenomic database obtained from *Spirulina* raceway ponds. (A) Metagenomic data obtained from the various fractions of the *Spirulina* samples are summarized. “ER” denotes a *Spirulina* metagenomic sample obtained from raceway ponds operated by Earthrise LLC. “S.S.” denotes a metagenomic sample collected from the nearby Salton Sea. Metagenomic assemblies obtained by Velvet and SPAdes programs are also summarized. The ultracentrifuged pellet from the Salton Sea extracellular fraction yielded no detectable RNA. (B) Snapshot of DNA sequencing reads from the ER cellular pellet mapped to the highly conserved bacterial *dnaK* gene in *A. platensis* NIES-39. The coverage plot shows ~100× coverage and is representative of the entire genome. Colored bars indicate mismatches between the sequencing reads and the reference sequence. (C) Conserved-domain search results for ER extracellular RNA contigs containing RNA replicase domain hits identified by HMMer 3.1. (D) Example of a DNA phage-like contig found in the ER extracellular DNA metagenome. Annotations shown were generated by the PHASTER online interface. Download FIG S5, EPS file, 2.6 MB. Copyright © 2017 Silas et al. 2017 Silas et al. This content is distributed under the terms of the Creative Commons Attribution 4.0 International license.

10.1128/mBio.00897-17.1 TEXT S1  Supplemental results. Download TEXT S1, DOCX file, 0.1 MB. Copyright © 2017 Silas et al. 2017 Silas et al. This content is distributed under the terms of the Creative Commons Attribution 4.0 International license.

Additionally, we sequenced CRISPR spacers from the cellular DNA using the amplification scheme from Fig. S3. The resulting “native” spacer repertoire exhibited characteristics similar to those of the previous dataset from store-bought *Spirulina*, and both store-bought and native spacers were included in subsequent searches (Fig. S6).

10.1128/mBio.00897-17.8 FIG S6  Sequencing of spacers from the cellular fraction of metagenomic samples from *Spirulina* raceway ponds. (A) Numbers of unique spacers recovered from type III-D and type III-B CRISPR arrays before and after clustering. The last two columns show Chao-1 estimates of species richness/diversity (57). The Chao-1 estimate corresponds to an approximate lower bound for the total number of unique spacers in the sample. The 95% confidence lower bound for the Chao-1 estimate has also been calculated. (B) Histogram of spacer frequencies before and after clustering. The last bin contains all sequences observed 100 times or more. (C) Histogram of spacer lengths for III-D and III-B spacers. (D) Saturation curves calculated for III-D and III-B spacer pools using the clustering algorithm described for panel A showing the number of sequence clusters obtained as progressively larger subsets of the spacer datasets are considered. Download FIG S6, EPS file, 2.1 MB. Copyright © 2017 Silas et al. 2017 Silas et al. This content is distributed under the terms of the Creative Commons Attribution 4.0 International license.

### An enigmatic source of the RT-associated CRISPR spacers from *Spirulina.*

**FIG 5:**
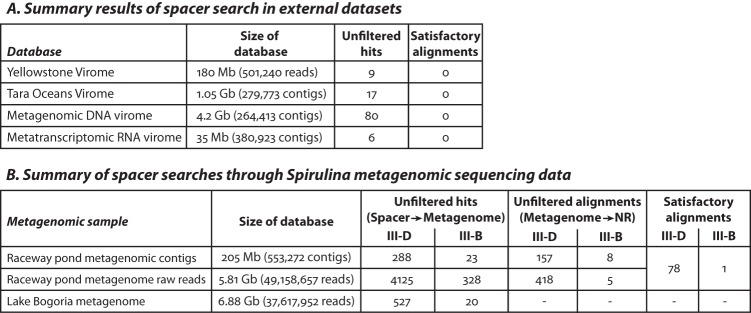
Summary of *Spirulina* spacer search attempts. (A and B) BLAST results matching ~2 million CRISPR spacers to reads and contigs from (A) various virome datasets and (B) *Spirulina* metagenomic data. The “unfiltered hits” columns show the number of matches returned at e-value stringency cutoffs based on the size of the database. The “Unfiltered alignments” column in panel B denotes the subsets of reads and contigs from the unfiltered hits that could in turn be identified in public protein sequence databases (NR) at the protein level. The “Satisfactory alignments” columns list the manually curated hits remaining after low-complexity and low-confidence matches were eliminated.

We attempted to identify a source for *Spirulina* spacers from the combined pool of ~2.5 × 10^6^ sequences representing >2 × 10^5^ clusters. Since searches through the *A. platensis* reference genomes and public (NCBI NR/NT) databases yielded very few matches (see Text S1), we shifted our focus to datasets that specifically aimed to collect bacteriophage sequences that were not present in NR/NT collections. Several such datasets were screened: a cyanobacterial virome sequenced from hot-spring microbial mats in Octopus Spring in Yellowstone National Park (29); all viral contigs from the Tara Ocean Viromes project, which contained many DNA bacteriophages of abundant marine cyanobacteria such as *Synechococcus* sp. (30); and a source-agnostic virome curated from a comprehensive analysis of thousands of metagenomic DNA samples (31, 32). None of the datasets yielded any conclusive identification of the *Spirulina* spacers (Fig. 5A). Next, to leverage the possibility that the type III-B CRISPR arrays were acquiring spacers exclusively from RNA, we also searched through an RNA virome constructed through a meta-analysis of metatranscriptomic contigs (unpublished) from the IMG/M system (33) containing RNA viral hallmark genes, including RNA-dependent RNA polymerase (RdRp) genes and other genes specific to RNA viruses. Again, we did not find any confident matches (Fig. 5A).

Next, leveraging the *Spirulina* metagenomes and viromes, we set out to find spacers that might have been derived from a sequence in the local environment which we could then potentially identify in external databases (also see Fig. S7, S8, and S9 and Text S1). While a few more hits for type III-D spacers were observed in the *Spirulina* metagenomic data relative to public sequence databases (Fig. 5B), the overwhelming majority still could not be matched. Notably, only matches to a single site in thymidylate synthase were observed among the type III-B spacers (Fig. S9B). Alignments of spacers to metagenomic reads that could be identified as homologues of known open reading frames (ORFs) are presented in Table S2 (https://figshare.com/s/16f85dbabc34e314a828). Finally, we also screened metagenomic sequencing data from Lake Bogoria but found only ~540 hits in a total search space of over 37 million reads. Almost all of these hits contained stretches of low-complexity sequence and were unlikely to represent genuine matches.

10.1128/mBio.00897-17.7 FIG S7  Spacer homology searches through public sequence databases. (A) Only 2 clustered spacers in a combined-sequence pool of >200,000 mapped to the reference genomes outside the native CRISPR arrays. The type III-B spacer mapping to the alcohol dehydrogenase gene is also contained in the CRISPR-B1 array in the *A. platensis* NIES-39 genome. (B) A Kmer-based mapping approach returns the expected number of random matches to the reference genome. (C) Only 2 of >200,000 clustered spacers can be identified by BLAST in the NCBI nucleotide collection (NT). (D) Example of two type III-B spacers combined by the greedy assembly algorithm. Download FIG S7, EPS file, 1.9 MB. Copyright © 2017 Silas et al. 2017 Silas et al. This content is distributed under the terms of the Creative Commons Attribution 4.0 International license.

10.1128/mBio.00897-17.9 FIG S8  Group I and group II self-splicing introns in *A. platensis*. (A) Reads from RNA and DNA sequencing of *Spirulina* cellular pellet mapping to predicted exon junction sequences from manually annotated group 1 and group 2 self-splicing introns in the *A. platensis* NIES-39 genome. An example of each is shown, with an RNA-Seq read spanning the predicted exon junction, and no support for the exon junction sequence being present as DNA in the sample. (B) Reads per kilobase per million (RPKM) values of the 30 most highly expressed genes in *A. platensis* NIES-39 as determined by sequencing of RNA from the *Spirulina* cellular pellet. Protein products of genes that were not annotated in the reference genome were identified by the presence of conserved domains using the NCBI CD-Search program (https://www.ncbi.nlm.nih.gov/Structure/bwrpsb/bwrpsb.cgi). Download FIG S8, EPS file, 1.7 MB. Copyright © 2017 Silas et al. 2017 Silas et al. This content is distributed under the terms of the Creative Commons Attribution 4.0 International license.

10.1128/mBio.00897-17.10 FIG S9  Spacer homology searches through a metagenomic database obtained from *Spirulina* raceway ponds. (A) Example of a type III-D spacer (~40 bp) that could be identified as a putative segment of a DNA methyltransferase-like gene in the *A. platensis* NIES-39 reference genome once it was mapped to a longer metagenomic read (~150 bp) derived from the ER extracellular DNA fraction that could be identified based on its coding potential. (B) The only type III-B spacer (~40 bp) that could be identified as a putative segment of a thymidylate synthase-like gene in the *A. platensis* NIES-39 reference genome once it was mapped to a longer metagenomic read (~150 bp) derived from the ER extracellular RNA fraction that could be identified based on its coding potential. (C) Example of a type III-D spacer (~40 bp) that could be identified as a putative segment of a DNA methyltransferase-like gene in the *Pseudomonas* sp. PH1b genome once it was mapped to a longer metagenomic read (~150 bp) derived from the ER extracellular RNA fraction that could be identified based on its coding potential. (D) Prophage-like region in the *Pseudomonas* sp. PH1b reference genome containing the spacer-matching sequence described in panel C. Download FIG S9, EPS file, 1.6 MB. Copyright © 2017 Silas et al. 2017 Silas et al. This content is distributed under the terms of the Creative Commons Attribution 4.0 International license.

In summary, screening of the various databases detailed here led to the identification of only a small proportion of spacers with protospacers detectable in the *Spirulina* spacer pool. Several protospacers were identified for the RT-lacking type III-D system, but only a single potential target was detected for the RT-associated III-B system. The low prevalence of detectable protospacers in the *Spirulina* metagenome points to an unknown origin of the great majority of the type III-D and (especially) type III-B protospacers in *A. platensis*.

### Several RT-linked CRISPR spacers appear to target DNA viruses.

We then broadened our search to all CRISPR spacers in published genomic datasets. To assess whether any CRISPR spacers were specific to any RNA phages, we aligned all unique sequences from known RNA phage species (see Table S3 at https://figshare.com/s/f373935a1da886040791) (34) to CRISPR spacers from published genomes (35). No RNA phage sequences showed significant similarity to any CRISPR spacer. Given the lack of detected protospacers at this stage, we expanded the search to include all spacers from CRISPR arrays associated with the RT-encoding loci (see Table S1 at https://figshare.com/s/3a8dab8ed7138922f693) to look for possible matches to the viral subset of the NR/NT database (NCBI taxid 10239) and against the prokaryotic database. Only a few matches were found among the 2,054 spacers (see Table S4 at https://figshare.com/s/72fccf4e2ad9503b5a0e), and most of these originated from DNA phages or predicted prophages. The CRISPR arrays containing the spacers with matches are scattered among different branches of the RT tree (Table S4, https://figshare.com/s/72fccf4e2ad9503b5a0e).

## DISCUSSION

The dynamic evolution of the CRISPR loci, both in their “hardware” (adaptation and effector enzymatic machinery) and “software” (spacer repertoires), provides a valuable opportunity to track virus-host coevolution (36). In this work, we analyzed the prevalent variants of type III CRISPR-Cas systems that contain reverse transcriptase domains and appear to acquire spacers from RNA molecules. To gain insight into the evolution and functions of these CRISPR-Cas systems, we examined the broad phylogeny of the RT-associated adaptation machineries and interrogated the resultant spacer repertoire in detail in a readily accessible bacterial species carrying such a locus (*Arthrospira platensis*).

Phylogenetic analysis of CRISPR-linked RTs revealed monophyly of the RT-Cas1 fusion, with some subsequent, lineage-specific fission events. The CRISPR-linked RTs are most closely related to group II intron-encoded RTs (26), suggesting that the initial recruitment of RT by the CRISPR-Cas system followed integration of a group II intron next to a type III *cas1* gene. This scenario is compatible with the detection of several cases of independent RT recruitment by narrow groups of archaea and bacteria, including bacterial type I-E. The apparent fixation of the association between the RT and CRISPR-Cas systems in each of these groups suggests that the group II intron RT is relatively easily adopted for spacer acquisition from RNA, and that fusion with Cas1 is not required. Nevertheless, the phylogenetic trees of the RT and Cas1 moieties of the RT-Cas1 fusion proteins are largely congruent, indicating that the two domains are involved in a functional interaction and tightly coevolve (although several isolated recombination events were detected). In contrast, there was little concordance between the phylogeny of CRISPR-linked RTs and the CRISPR type classification (largely based on the sequence and arrangements of CRISPR effector modules [7]), suggesting that the RT-Cas1 adaptation module is functionally autonomous, is disseminated via horizontal gene transfer, and can combine and function (perhaps even in *trans*) with diverse type III effector modules.

The various sources of commercial *Spirulina* provided a rich sampling of environmentally derived CRISPR spacers for both RT-linked (type III-B) and non-RT-linked (type III-D) CRISPR arrays in the same host genome. Both repertoires were remarkably diverse, rivaling the host genome in sequence content. Furthermore, while the spacer repertoire of the RT-lacking III-D system seemed to show signs that it was approaching saturation, the repertoire of the RT-containing III-B systems did not. Consistent with our relative ignorance of the natural populations of genome parasites for *A. platensis* (or for most microbes), we were able to identify protospacers for only a small fraction of spacers. Almost all of the identified spacers were from the type III-D arrays, making the origin of the spacers in the RT-linked type III-B loci particularly enigmatic. These findings indicate that the type III-B system might acquire spacers from a nucleic acid sequence pool distinct from the source of type III-D spacers. Such a distinct pool could potentially include viruses that are encountered only transiently by *A. platensis* in its natural environment. As a few intriguing (and unsupported) possibilities, the type III-B spacers could be generated from a pool of autonomously replicating RNA (37), by a random process (38), or from an as-yet-unknown pool of environmental nucleic acids.

Type III CRISPR-Cas systems can target RNA (17–24), and, given the demonstrated ability of the RT-containing type III-B system to also integrate spacers from RNA via reverse transcription (16), it appeared an interesting possibility that these variants of CRISPR-Cas could provide adaptive immunity against RNA-based parasites, such as RNA bacteriophages. However, no natural examples of CRISPR immunity against RNA phage have been described so far, nor have CRISPR spacers mapping to an RNA virus been identified in any prokaryote. In this work, we were unable to identify any spacers derived from RNA viruses despite extensive metagenomic sampling of the extracellular environment. Such efforts could be stymied by the relatively small number of currently characterized RNA phage (34, 39).

Type III CRISPR-Cas systems can also mediate defense against transcriptionally active DNA phages (40, 41). The ability to acquire spacers from RNA could help direct CRISPR immunity toward phage transcripts that are expressed early in the infection cycle, and toward highly expressed phage genes. Spacer acquisition from RNA could also enable sampling of phage genomes that could be protected from DNA-targeting CRISPR-Cas nucleases through DNA modification (42). Furthermore, a transcription-dependent targeting mechanism could allow microbes to tolerate lysogenic viruses integrated stably in the host genome and yet have a ready response upon prophage induction (20). A broad search of the CRISPR spacers linked to RTs in all available prokaryotic genomes against all nonredundant protein records indeed yielded several matches to DNA phage-like sequences. These results suggest that the RT-mediated mechanism of spacer acquisition by CRISPR-Cas systems may be used at least in part to provide defense against transcriptionally active DNA-based parasites.

The origin of the CRISPR spacers is a general, unresolved problem. A substantial majority of the spacers in most CRISPR arrays (over 90% on average, according to the latest comprehensive survey [43]) have no detectable matches in known sequences. However, to our knowledge, very few spacer repertoires and associated metagenomes have been sequenced to the depth reported here for *A. platensis*. The extreme diversity of spacers, especially in the RT-linked arrays, combined with the effective lack of detectable protospacers, indicates that a key element is missing from our picture of CRISPR-Cas biology: the sources of the spacers remain enigmatic. Part of the problem could stem from the high rate of spacer loss and mutation and, likely, an even higher rate of mutational escape of viruses, which result in accumulation of mismatches between a spacer and its cognate protospacer, rendering the latter unrecognizable. Nevertheless, another cause of the lack of detectable protospacers is likely to be the existence of a virtually untapped pool of mobile genetic elements that, at any given time, are not represented in a given environment (10). This paucity of spacer matches is conceivably explained by the vastness and diversity of viromes combined with the rapid evolution of the (proto)spacers (43). Longitudinal studies on metagenomes could help characterize that vast and currently enigmatic sequence pool (25, 36).

## MATERIALS AND METHODS

### Prokaryotic genome database and open reading frame annotation.

Archaeal and bacterial complete and draft genome sequences were downloaded from the NCBI FTP site (ftp://ftp.ncbi.nlm.nih.gov/genomes/all/) in March 2016. For incompletely annotated genomes (coding density of less than 0.6 coding DNA sequences [CDS] per kbp), the existing annotation was discarded and replaced with a Meta-GeneMark 1 (44) annotation with the standard model MetaGeneMark_v1.mod (heuristic model for genetic code 11 and GC 30). Altogether, the database includes 4,961 completely sequenced and assembled genomes and 43,599 partially sequenced genomes. Profiles for RT families (cd01651, pfam00078, and COG3344) that are included in the NCBI CDD database (45) were used as queries for a PSI-BLAST search (e-value = 1e−4) to identify RT homologs. The RT genes were used as a seed to identify defense islands as described previously (46). All ORFs within loci were annotated using RPS-BLAST searches with 30,953 profiles (COG, pfam, cd) from the NCBI CDD database and 217 custom Cas protein profiles (7). The CRISPR-Cas system (sub)type identification for all loci was performed using previously described procedures (7).

### Sequence clustering, alignment, and phylogenetic analyses.

To construct a nonredundant, representative RT sequence set, sequences were clustered using the NCBI BLASTCLUST program (ftp://ftp.ncbi.nih.gov/blast/documents/blastclust.html) with a sequence identity threshold of 90% and length coverage threshold of 0.9. Short fragments or disrupted sequences were discarded. Multiple alignments of protein sequences were constructed using MUSCLE (47). Sites with gap character fraction values of >0.5 and homogeneity values of <0.1 were removed from the alignment. Phylogenetic analysis was performed using the FastTree program (48), with the WAG evolutionary model and the discrete gamma model with 20 rate categories. The same program was used to compute SH (Shimodaira-Hasegawa)-like node support values.

### High-throughput sequencing of CRISPR spacers.

This method is a slight modification of a previously published protocol for CRISPR spacer sequencing (16); we have provided the protocol in its entirety for completeness, retaining relevant text from the original protocol. CRISPR spacers were amplified by PCR from 1 to 2 ng genomic DNA per μl PCR mix using primers anchored in the various CRISPR repeat sequences. The primers used for type III-D CRISPR arrays were as follows: SS-4F, CGACGCTCTTCCGATCTNNNNNCTTGCGGGGAATTGGTAGGG; SS-4R, ACTGACGCTAGTGCATCAAATTCCCCGCAAGGGGACGG; SS-5F, CGACGCTCTTCCGATCTNNNNNCCAATTCCCCGCAAGGGGAC; SS-5R, ACTGACGCTAGTGCATCATGCGGGGAATTGGTAGGGTC; SS-8F, CGACGCTCTTCCGATCTNNNNNCCAATTCCCCGTCAGGGGAC; and SS-8R, ACTGACGCTAGTGCATCAGACGGGGAATTGGTAGGGTT. The primers used for type III-B CRISPR arrays were as follows: SS-19F, CGACGCTCTTCCGATCTNNNNNTAACTTTCARAGAAGTYTAA; SS-19R, ACTGACGCTAGTGCATCATGAAAGTTAAACGTATGGAA; SS-20F, CGACGCTCTTCCGATCTNNNNNCGACTTTCAAAGAAGTCTCA; SS-20R, ACTGACGCTAGTGCATCATGAAAGTCGAACGTATGGCA; SS-51F, CGACGCTCTTCCGATCTNNNNNTTCTYTGAAAGTTAAACGTA; and SS-51R, ACTGACGCTAGTGCATCATTTAACTTTCARAGAAGTTT. F and R denote forward and reverse primers. Primers with the same numeric code were used together. Letters correspond to IUPAC nucleic acid notation. CRISPR repeat matching regions are underlined.

Sequencing adaptors were then attached in a second round of PCR with 0.01 volumes of the previous reaction mixture as the template, using AF-SS-44:55 (CAAGCAGAAGACGGCATACGAGATNNNNNNNNGTGACTGGAGTTCAGACGTGTGCTCTTCCGATCACTGACGCTAGTGCATCA) and AF-KLA-67:74 (AATGATACGGCGACCACCGAGATCTACACNNNNNNNNACACTCTTTCCCTACACGACGCTCTTCCGATCT), where the (N)_8_ barcodes correspond to Illumina TruSeq HT indexes D701 to D712 (reverse complemented) and D501 to D508, respectively. Template-matching regions in primers are underlined. Phusion High-Fidelity PCR master mix with HF buffer (Fisher Scientific) was used for all reactions. Cycling conditions were as follows: 98°C for 1 min; 2 cycles of 98°C for 10 s (60°C for 20 s for primer pairs AF-SS-4, AF-SS-5, and AF-SS-8; 44°C for 20 s for primer pair AF-SS-19; 50°C for 20 s for primer pair AF-SS-20) and 72°C for 30 s; 18 cycles of 98°C for 15 s (70°C for 15 s for primer pairs AF-SS-4, AF-SS-5, and AF-SS-8; 63°C for 15 s for primer pair AF-SS-19; 66°C for 15 s for primer pair AF-SS-20) and 72°C for 30 s; and 72°C for 9 min for round 1, and 98°C for 1 min; 2 cycles of 98°C for 10 s, 54°C for 20 s, and 72°C for 30 s; 4 cycles of 98°C for 15 s, 70°C for 15 s, and 72°C for 30 s; and 72°C for 9 min for round 2. The dominant amplicons (250 to 275 bp) containing a mixture of spacer sequences were excised following agarose electrophoresis (3%, 4.2 V/cm, 2 h) of round 2 PCR products. Libraries were quantified by Qubit and sequenced with Illumina MiSeq v3 kits (150 cycles for read 1; 8 cycles for index 1; 8 cycles for index 2).

Spacers were trimmed from reads using a custom python script and were considered identical if they differed by only 1 nucleotide. Protospacers were mapped using Bowtie 2.0 (–very-sensitive-local alignments). These methods preserve strand information.

### Preparation of cellular and extracellular fractions from *Spirulina* samples.

Prepackaged *Spirulina* samples were purchased from various vendors as described for Fig. 1. A similar probiotic blend containing a variety of plant matter but lacking *A. platensis* was also tested as a negative control for CRISPR spacer amplification.

*Spirulina* metagenomic samples were collected in 50-ml polypropylene centrifugal tubes (Corning) from open-air raceway ponds operated by Earthrise LLC, Calipatria, CA, and were transported on ice to our laboratory for processing the same day (without freezing). *Spirulina* at these farms is grown in an interconnected network of open-air ponds, containing approximately 1 million liters of water seeded with inorganic nutrients and injected with carbon dioxide to support the high growth rate of the enriched cyanobacterial culture. The culture is kept in continuous circulation between ponds using paddle wheels and is maintained continuously from April through October. Growth of unwanted “weed algae” is prevented by raising the pH of the culture to leverage the rare ability of *A. platensis* to grow in alkaline environments.

Cyanobacteria were pelleted from ~120 ml of pond water by centrifugation at 4,000 × *g* for 1 h (Beckman Allegra X-15R) (4°C). The supernatant was then divided into four 30-ml polypropylene high-speed centrifugal tubes (Nalgene Oak Ridge) and subjected to a preclearing spin at 12,000 × *g* for 1 h (Avanti J-25I centrifuge with JA-17 rotor; Beckman Coulter, Inc.) (4°C). The cleared sample was further subdivided into 15-ml open-top Polyallomer tubes (Seton), and extracellular material was collected by ultracentrifugation at 200,000 × *g* for 16 h (Optima XE-90 Ultracentrifuge with SW41 Ti rotor; Beckman Coulter, Inc.) (4°C) with and without prior filtration through a 0.45-µm-pore-size Polysulfone membrane (Pall Corp.).

### Nucleic acid extraction from *Spirulina.*

The cyanobacteria marketed as *Spirulina* are typically subjected to cold compression into pellets sold as food supplements, or flash-dried and sold as a powder intended to be mixed into kitchen recipes. Genomic DNA would be expected to remain intact through the packaging process, which eschews heat and mechanical granulation. Genomic DNA was extracted from *Spirulina* grocery store samples and metagenomic cellular fractions as previously described (49). RNA was extracted from *Spirulina* grocery store samples and metagenomic cellular fractions using a combined TRIzol/RNeasy method (16).

DNA extractions from metagenomic extracellular fractions were performed using a modified SDS/protease K method. Briefly, pellets were resuspended in 100 μl of lysis buffer (10 mM Tris, 20 mM EDTA, 50 μg/ml protease K, 0.5% SDS) and incubated at 56°C for 1 h. DNA was precipitated with the addition of isopropanol at up to 50% of the total volume. DNA pellets were washed with 70% ethanol and resuspended in 10 mM Tris (Qiagen) (pH 8.5). Metagenomic extracellular DNA samples were prepared by two methods: with and without RQ1 RNase-Free DNase (Promega) pretreatment of the ultracentrifuged pellet (per the manufacturer’s instructions). The data obtained through the two methods were similar.

RNA samples from metagenomic extracellular fractions were prepared by two methods: with and without RQ1 DNase pretreatment of the ultracentrifuged pellet. RNA was extracted from the pellets using TRIzol (Life Technologies, Inc.) per the manufacturer’s instructions. Purified RNA was treated with RQ1 RNase-Free DNase, which was subsequently removed by extraction performed with a 1:1 mixture of acidified phenol (Ambion) and chloroform (Fisher Scientific), followed by an extraction performed with chloroform and precipitation of RNA from the aqueous phase through the addition of ethanol at up to 70% of the total volume. RNA pellets were washed with 70% ethanol and resuspended in RNase-free water (Qiagen).

### DNA sequencing of *Spirulina* samples.

Genomic DNA extracted from *Spirulina* grocery store samples and metagenomic cellular fractions was prepared for high-throughput sequencing using a Nextera DNA Library Prep kit (Illumina) according to the manufacturer’s instructions.

### RNA sequencing of *Spirulina* samples.

Three different methods were employed for RNA sequencing. The first was described previously (16); this method provides unbiased sequencing, especially of shorter RNA fragments that may be missed by other protocols. The second was carried out according to the instructions provided with a SMARTer Stranded RNA-Seq kit (Clontech); this method was especially useful in generating libraries from low-concentration RNA samples (e.g., *Spirulina* metagenome extracellular RNA fractions). The third method was developed previously in our laboratory for detecting low-abundance RNA species. Up to 100 ng of RNA was diluted to 6 μl in RNase-free water and incubated at 90°C for 1 min and then at 70°C for 4 min and was transferred to ice for 2 min. A 13.5-μl volume of reverse transcription master mix (4 μl 5x First Strand buffer, 2 μl 0.1 M dithiothreitol, 2 μl 10 mM deoxynucleoside triphosphate [dNTP] mix, 1 μl RNase-Out, 1.5 μl Superscript II reverse transcriptase, 3 μl RNase-free water [all components from Life Technologies, Inc.]) was added to each sample, and the mixture was incubated at 42°C for 30 min. A 0.5-μl volume of 100 ng/μl exonuclease-resistant random hexamers (Fisher Scientific) was then added, and the reaction mixtures were incubated at 25°C for 2 min and at 42°C for an additional 60 min. The reaction was terminated by heating to 95°C for 5 min. Subsequently, 127.5 μl of multiple-displacement amplification (MDA) master mix (6 μl of 25 mM dNTP mix [Roche], 15 μl of 10x Phi29 DNA polymerase reaction buffer [NEB], 7.5 μl of 1 μg/μl exonuclease-resistant random hexamers, 7.5 μl of 0.1 M dithiothreitol, 93 μl of RNase-free water) was added to each reaction, and the mixture was split into 3 tubes at 47.5 μl each and incubated at 95°C for 5 min and then at 4°C during the addition of 2.5 μl of Phi29 DNA polymerase (NEB) to each tube. The reaction mixtures were incubated at 30°C for 6 to 8 h. The MDA product was purified using Zymo DNA clean-and-concentrator columns and prepared for sequencing using a Nextera DNA Library Prep kit.

RNA from cellular fractions of the *Spirulina* metagenome was sequenced using all three methods. RNA samples from extracellular fractions (with and without filtration using 0.45-µm-pore-size filters and with and without DNase treatment) were sequenced using only the SMARTer Stranded and MDA methods as there was not enough input material for the small-RNA sequencing method described in reference 16. The samples processed via the SMARTer Stranded protocol were prepared with and without the built-in RNA fragmentation step in an effort to capture shorter RNA fragments.

### Computational analyses of *Spirulina* datasets.

CRISPR spacers were trimmed from high-throughput sequencing reads and clustered to account for sequencing errors, with 1 allowed mismatch on the Illumina MiSeq platform and 2 allowed mismatches on the Illumina HiSeq platform. All searches for sources of spacer sequences were carried out using NCBI blast package 2.2.25, with a culling limit of 1 and an empirically determined e-value cutoff for each dataset to minimize false negatives as reported in the text. Blast databases were formatted using formatdb. Preformatted nucleic acid datasets (NT; nucleotide collection; posted 9 February 2013) and protein datasets (NR; ll nonredundant GenBank CDS translations plus PDB plus SwissProt plus PIR plus PRF, excluding environmental samples from whole-genome sequencing [WGS] projects; posted 13 March 2015) were obtained from NCBI. HMM searches for RdRP-related ORFs were carried out using HMMER 3.1 (50), and phage-like contigs were identified using the PHASTER online interface (51). Contigs were assembled using both Velvet 1.1.07 (velveth run with a maximum Kmer length of 31 and velvetg with a minimum contig size of 200) (52) and SPAdes 3.7.1 (53) in metagenomic mode. Alignments of metagenomic sequencing reads and CRISPR spacers to the reference genome(s) were carried out using bowtie 2.2.6 using the –very-sensitive-local option. The bedtools 2.25.0 merge program was used to collapse redundant alignments on the basis of the location. Custom python scripts were written for “greedy” spacer assembly, clustering of spacer sequences, translation of putative ORFs in metagenomic contigs, and curation of BLAST result files.

### Metagenomic protospacer analysis.

For broader metagenomic searches, the CRISPRfinder (54) and PILER-CR (55) programs were used with default parameters to identify CRISPR arrays found in Cas7f and TnsA/TnsD loci. The MEGABLAST program (56) (word size, 18; otherwise, default parameters) was used to search for protospacers in the virus subset of NR database and the prokaryotic genome database. We considered only those matches with 95% identity and 95% length coverage or better with respect to the NR database.

### Accession number(s).

Sequencing data have been deposited at SRA (SRP107814).
