# Supplementary material for: On the Origin of Reverse Transcriptase-Using CRISPR-Cas Systems and Their Hyperdiverse, Enigmatic Spacer Repertoires
Source: mBio. 2017 Jul 11;8(4):e00897-17. doi: 10.1128/mBio.00897-17 (PMC5513706; doi:10.1128/mBio.00897-17)
Supplement: TEXT S1 [file mbo003173378s1.docx]

**Supplemental Results**

***Spirulina* spacer searches through available sequence databases**

We wondered if we could ascertain the source sequences of the *Spirulina* spacers in previously generated datasets. Since RT-Cas1 associated type III-B arrays would be expected to acquire spacers from RNA, our initial hypothesis was that such spacers could be derived from host transcripts and might possess a function in gene regulation. However, we found no convincing matches in the *A. platensis* reference genomes for our spacers outside of the various CRISPR arrays. Whole spacers mapped to the reference using bowtie 2.0 or BLAST failed to identify any matches outside of the CRISPR arrays longer than 30 bp, except for a 41 bp match to an alcohol/acetaldehyde dehydrogenase gene in *A. platensis* NIES-39, and a 43 bp match to a DNA methyltransferase gene in *A. platensis* PCC8005 (**Figure S7A**). The 41 bp sequence in the alcohol/acetaldehyde dehydrogenase gene is also present as a spacer in the CRISPR-B1 array in the reference genome. No other spacers from the combined pool could be identified by this approach. To assess whether this failure could be attributed to a high proportion of polymorphisms in the natural *Spirulina* population, we used a kmer-based mapping approach instead, and attempted to map segments of various lengths tiled across each spacer back to the reference genomes. This approach yielded a proportion of mapped segments that would be expected by random chance (**Figure S7B**).

The *A. platensis* genome contains many mobile elements, notably group I and group II self-splicing introns. These introns would, upon splicing out of their parent transcripts, yield novel exon-junctions that are not present in the reference genome at the DNA level. As this could potentially mitigate any lethal consequences of autoimmunity, we wondered if any spacers mapped to such putatively spliced sequences. We compiled a list of ~150 group I and group II intron-like sequences in the *A. platensis* reference genome, and separately confirmed that some of these introns were indeed spliced to generate the predicted RNA sequence junctions that were absent in DNA in *Spirulina* populations (**Figure S8A**). Surprisingly, we found that intron-associated reverse transcriptases were among some of the highest expressed genes in these cyanobacteria (**Figure S8B**). However, no spacers mapping to these junctions were found by BLAST; we repeated the searches with the minimum possible penalties for gap opening and extension to allow for a small amount of human error in predicting the intron splice sites, but did not obtain any hits.

Next, we attempted to identify spacers by BLAST against public sequence datasets (NT) with low e-value stringency cutoffs to avoid a circumstance where bonafide hits with weak homology to their sources were ignored. The search returned a number of low-complexity sequences. The only convincing matches that were retrieved in our searches were spacers corresponding to ~40 bp portions of an IS200 family cyanobacterial transposase, and a cyanobacterial gene similar to an alcohol/acetaldehyde dehydrogenase (**Figure S7C**, also see **Figure S7A**). All but these two of the *Spirulina* spacers remained unidentified by this approach.

Bacterial populations co-evolve with their parasites such as phage and plasmids ([36](#_ENREF_37)), and these invasive nucleic acids are often highly host-specific. We wondered whether portions of the genomes of putative parasites of *A. platensis* could be reconstructed by assembling the CRISPR spacers. In an attempt to assemble the spacer sequences themselves into longer “protospacer contigs” that might be more easily identified, we used a “greedy” assembly algorithm that assembles any sequences with a greater than 8 bp overlap. While we were able to re-construct some complex sequences in this way (**Figure S7D**), we were unable to identify any ORF-like regions the assemblies in public protein sequence datasets (NR).

***Spirulina* spacer searches through ‘raceway pond’ metagenomic databases**

We first attempted to discern whether our datasets contained information about parasitic RNA or DNA in *Spirulina*. To aid in identification of sequences in the extracellular fraction, we assembled reads from each sample (cellular DNA, cellular RNA, extracellular DNA, and extracellular RNA) into contigs using two commonly used assembly algorithms, velvet and spades ([52](#_ENREF_53), [53](#_ENREF_54)). We obtained many contigs from each dataset (**Figure S5A**), which were then translated into protein sequence to take advantage of potentially greater conservation of genetic information at the protein level. Although the first observation of a cyanophage infecting *A. platensis* was only made in 2012 ([58](#_ENREF_58)), we hoped that other phages capable of infecting these cyanobacteria might share conserved sequence features of core viral proteins with known viruses. However, simply searching translated contigs against phage databases could generate false positives due to non-viral genes being identified as phage derived sequence in the absence of their “true” match in the reference set. We therefore used more conservative methods to identify phages in the extracellular DNA and RNA datasets. Replicating RNAs such as RNA phage typically encode a mechanism to direct RNA synthesis from RNA templates ([59](#_ENREF_59)). Proteins that enable RNA replication (known as replicases) are therefore considered “signature” genes for RNA viruses with no DNA stage in their lifecycle. We processed translated contigs from all *Spirulina* datasets using hidden markov models ([50](#_ENREF_51)) corresponding to 18 known replicases, and found several hits for the phage-related RdRP model (**Figure S5C**). Even though we searched through all our metagenomic contigs, RdRP hits were only found in sequences in the extracellular RNA datasets, giving us confidence in the validity of our methods to find RNA viruses. Identifying DNA phage is more difficult since they do not contain any “signature” genes; however, several tools have been developed to identify DNA phage-like sequences in metagenomic datasets. We used the online PHASTER interface ([51](#_ENREF_52)) and identified several promising phage derived contigs (**Figure S5D**), further validating our enrichment and sequencing approach.

To identify putative metagenomic sources of *Spirulina* spacers, we compared spacers with metagenomic contigs generated by the spades and velvet assemblers from the *Spirulina* raceway pond datasets. Among over 500,000 contigs, only about 300 were hit by spacers below a 10^-6^ e-value threshold. Not all of the hits were convincing, with many matches spanning only a portion of the spacers, and others containing stretches of low complexity sequence such as short repeats or homopolymer runs. This suggested that our criteria were not inadvertently excluding bonafide spacer-contig matches. None of the ~300 contigs could be identified as phage-like sequence using HMM models of RdRPs for RNA viruses, or the PHASTER algorithm for DNA viruses.

We then attempted to identify putative ORFs in these ~300 contigs by translated nucleotide BLASTx against the NR protein database with a loose 10^-3^ e-value cutoff. We chose to search against the entire non-redundant protein database instead of a virus-specific sequence collection in order to avoid forced matches between contigs and virus-like sequences that would otherwise have been superseded by a better match to a non-viral protein in the larger dataset. Roughly half (~150) contained ORFs that could be identified; curiously, many of these ORFs most closely resembled various proteins in the *Arthrospira* genome (**Figures S9A, B**). Since we were previously unable to map spacers to the *Arthrospira* reference genomes at the nucleotide level, these matches to “self” genes at the protein level were presumably missed earlier in the absence of metagenomic data due to the highly polymorphic nature of the *Spirulina* population (see **Figure S5B**). However, there were also several “trivial” matches to contigs containing portions of CRISPR arrays from the reference genomes, which were identified as self-sequence due to the presence of neighboring ORFs, or due to mis-annotation of parts of the CRISPR arrays as ORFs. Finally, some metagenomic sequences hit by spacers could also be identified as sequences resembling genes in other bacteria (**Figure S9C**). The *Pseudomonas* PH1b DNA methyltransferase shown in Figure S9C appears to reside in a putative prophage (**Figure S9D**). This could indicate that the spacer match to this gene reflects its derivation from a phage DNA methyltransferase.

Next, we compared spacers against raw sequence data from the *Spirulina* pond metagenome. Among almost 50 million sequences, only a few over 4000 appeared to be hit by spacers. As before, many matches spanned only a portion of the spacers or contained low complexity sequence, suggesting that a 10^-6^ e-value cutoff was not unduly restrictive. These raw reads and assemblies were too short to be probed by PHASTER or HMM methods, and only ~400 returned matches in the NR protein database by BLASTx. In addition to the many spurious matches, most satisfactory hits were again to proteins from *Arthrospira* and other bacterial genomes. It is possible that some of these sequences were derived from bacterial parasites that had been previously observed in reference sequencing datasets but subsequently mis-classified as genomic sequence. However, we cannot definitively ascribe a viral or parasitic origin to our matches in the absence of other corroborating evidence.

**Supplemental References**

58. Jacquet S, Zhong X, Parvathi A, Ram ASP. 2013. First description of a cyanophage infecting the cyanobacterium Arthrospira platensis (Spirulina). Journal of Applied Phycology 25:195-203.

59. Blumenthal T, Carmichael GG. 1979. RNA replication: function and structure of Qbeta-replicase. Annu Rev Biochem 48:525-48.
